# Research progress of plant-derived natural products in thyroid carcinoma

**DOI:** 10.3389/fchem.2023.1279384

**Published:** 2024-01-10

**Authors:** Qiujing Du, Weidong Shen

**Affiliations:** ^1^ The Affiliated Jiangyin People’s Hospital of Nantong University, Jiangyin, China; ^2^ Third Hospital of Shanxi Medical University, Shanxi Bethune Hospital, Shanxi Academy of Medical Sciences, Tongji Shanxi Hospital, Taiyuan, China

**Keywords:** natural product, thyroid carcinoma, treatment challenges, thyroid carcinoma survivors, redifferentiation, drug resistance

## Abstract

Thyroid carcinoma (TC) is a prevalent malignancy of the endocrine system, with a notable rise in its detection rate in recent decades. The primary therapeutic approaches for TC now encompass thyroidectomy and radioactive iodine therapy, yielding favorable prognoses for the majority of patients. TC survivors may necessitate ongoing surveillance, remedial treatment, and thyroid hormone supplementation, while also enduring the adverse consequences of thyroid hormone fluctuations, surgical complications, or side effects linked to radioactive iodine administration, and encountering enduring physical, psychosocial, and economic hardships. *In vitro* and *in vivo* studies of natural products against TC are demonstrating the potential of these natural products as alternatives to the treatment of thyroid cancer. This therapy may offer greater convenience, affordability, and acceptability than traditional therapies. In the early screening of natural products, we mainly use a combination of database prediction and literature search. The pharmacological effects on TC of selected natural products (quercetin, genistein, apigenin, luteolin, chrysin, myricetin, resveratrol, curcumin and nobiletin), which hold promise for therapeutic applications in TC, are reviewed in detail in this article through most of the cell-level evidence, animal-level evidence, and a small amount of human-level evidence. In addition, this article explores possible issues, such as bioavailability, drug safety.

## Graphical Abstract

**Figure:**
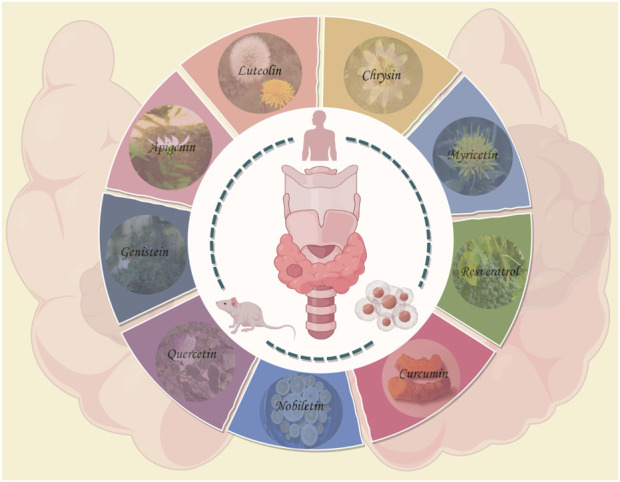
The pharmacological effects on TC of selected natural products (quercetin, genistein, apigenin, luteolin, chrysin, myricetin, resveratrol, curcumin and nobiletin), which hold promise for therapeutic applications in TC, are reviewed in detail in this article through most of the cell-level evidence, animal-level evidence, and a small amount of human-level evidence. All plant images, including herbs, are from Wikipedia. This image is drawn by Figdraw, and the image number for which the copyright is granted is OSTTP7c868.

## 1 Introduction

Thyroid carcinoma (TC) is a prevalent malignancy, with a notable increase in its detection rate over recent decades. According to the categorization outlined in the 5th edition of the World Health Organization (Baloch et al., 2022), endocrine tumors originating from follicular cells in the thyroid gland are primarily classified as follicular carcinoma, papillary carcinoma, eosinophilic carcinoma, high-grade follicular-derived carcinoma, etc. High-grade follicular-derived carcinoma mainly includes differentiated high-grade carcinoma, poorly differentiated carcinoma, and undifferentiated follicular-derived carcinoma, and medullary thyroid cancer is a unique type as thyroid C-cell-derived tumor. The prevalence of papillary thyroid carcinoma (PTC), the most commonly occurring type of thyroid cancer, has exhibited an upward trend over the past 30 years, particularly in Northern America, Asia, Europe, and other regions (SEER*Explorer: an interactive website for SEER cancer statistics [Internet], 2023). In the last decade, this increase has plateaued. PTC generally presents a positive prognosis, with a five-year survival rate surpassing 99% irrespective of gender (Bray et al., 2018). However, the prognosis for advanced PTC is typically unfavorable (Zhong et al., 2019). Medullary thyroid carcinoma (MTC) is a relatively uncommon form of thyroid cancer, representing approximately 1%–2% of all cases (Angelousi et al., 2022). However, it is responsible for a significant proportion of thyroid cancer-related deaths, accounting for approximately 13% of all thyroid cancer-related deaths. The majority of MTC cases are sporadic, and 25 percent are associated with genetic mutations in the RET proto-oncogene (Randle et al., 2017). Anaplastic thyroid cancer (ATC), as undifferentiated thyroid cancer, is a rare but highly aggressive malignancy of the thyroid. It constitutes a mere 2% of all thyroid cancers, yet its aggressive nature is evident as it accounts for 15%–50% of patients with distant metastases (Haddad et al., 2022). The prognosis for patients with anaplastic thyroid carcinoma is exceedingly unfavorable, with a median survival time less than 4–12 months (Jannin et al., 2022) and a five-year disease-free survival rate approaching 0% (Ramirez-Moya and Santisteban, 2021). Standard therapies for certain types of thyroid carcinomas, such as differentiated thyroid carcinomas (DTCs) and medullary thyroid carcinomas, as well as virtually all cases of ATC, demonstrate limited efficacy, frequently leading to metastasis to distant sites (Naoum et al., 2018). Consequently, there remains a pressing need to introduce more dependable treatment modalities for thyroid cancer.

Natural products (NPs) can be broadly defined as a group of small molecules from the environment, mostly genetically encoded and produced by secondary metabolic pathways, and NPs and their molecular frameworks are becoming an important source for the exploration of medicinal chemistry and therapeutic compounds (Pye et al., 2017). From 1981 to 2019, a significant proportion of anticancer drugs approved by the Food and Drug Administration (FDA) consisted of NPs and their derivatives, accounting for approximately 75% of the total (Newman and Cragg, 2020). Notable examples include paclitaxel, a broad-spectrum anti-cancer drug approved in the previous century, pyrotinib, recently approved for breast cancer treatment, and sintilimab, approved for Hodgkin lymphoma treatment. Consequently, NPs remain pivotal in the quest for novel agents and are considered the optimal selection for active templates. NPs are of interest due to their interesting biological activity and chemical structure (Ahmed et al., 2022). Over the years, natural product-based medicines have made significant progress in the treatment of human diseases and have shown great potential, which is one of the reasons why researchers around the world remain enthusiastic about it (Newman, 2022). Contemporary computation omics technology can help researchers effectively identify drug candidates and develop clinical drugs from too many molecules produced in nature (Rodrigues et al., 2016; Mullowney et al., 2023), encompassing genomics, transcriptomics, proteomics, metabolomics, bioinformatics, and integrative omics (Zhang HW. et al., 2021a).

In *in vitro* and *in vivo* studies of thyroid cancer, NPs have shown good therapeutic potential. Numerous studies have demonstrated that various natural compounds derived from food and herbs possess the ability to inhibit TC, including inhibiting the occurrence and development of TC (Yu et al., 2013; Hong et al., 2021; Lu et al., 2022), promoting redifferentiation of poorly differentiated TC (Lakshmanan et al., 2015; Zhang L. et al., 2021b), alleviating TC drug treatment resistance (Li et al., 2018; Bian et al., 2020; Celano et al., 2020), and so on. Previously, my research group published basic research on tangeretin and nobiletin. Based on the good experimental results, we are full of interest in plant-derived NPs, especially those distributed in traditional Chinese medicine. I made a preliminary prediction of disease-related active ingredients through the traditional Chinese medicine systems pharmacology database (TCMSP) and analysis platform database (Ru et al., 2014), and combined with the literature search results, to select nine NPs for review. Details are in Table 1. The pharmacological effects on TC of selected NPs, which hold promise for therapeutic applications in TC, are reviewed in detail in this article through most of the cell-level evidence, animal-level evidence, and a small amount of human-level evidence.

**TABLE 1:** Natural product properties in the TCMSP database.

Name	Pubchem CID	OB (%)	DL	Caco-2	MW
Quercetin	5280343	46.43	0.28	0.05	302.25
Genistein	5280961	17.93	0.21	0.43	270.25
Apigenin	5280443	23.06	0.21	0.43	270.25
Luteolin	5280445	36.16	0.25	0.19	286.25
Chrysin	5281607	22.61	0.18	0.70	254.25
Myricetin	5281672	13.75	0.31	−0.15	318.25
Resveratrol	445154	19.07	0.11	0.80	228.26
Curcumin	5281767	5.15	0.41	0.50	368.41
Nobiletin	72344	61.67	0.52	1.05	402.43

## 2 Thyroid cancer treatment and challenges

Thyroidectomy and radioactive iodine therapy have become the mainstay of treatment for TC. Patients with DTC without local progression and local/distant metastases can usually achieve better outcomes with surgery and radioactive iodine (RAI) therapy (Liu et al., 2023). Nonetheless, the rates of structural recurrence (locoregional or distant metastases) in low-risk, intermediate-risk, and high-risk patients are 3%–13%, 21%–36%, and 68%, respectively, and patients are classified by the American Thyroid Association risk stratification criteria (Coca-Pelaz et al., 2023).For patients with unresectable or metastatic DTC, RAI therapy is considered the primary therapeutic approach. Only one-third of patients achieve complete remission, and those outside of it become RAI refractory (RAIR) and have a poor overall prognosis, which is an unavoidable problem in the current medical management of the disease (Jin et al., 2018). 5%–15% of DTCs and 50% of metastatic DTCs progress to RAIR, and overall survival is significantly reduced, less than 10% at 10 years (Lorusso et al., 2023). Treatment options for symptomatic, rapidly progressing, inoperable locally advanced/extensive metastatic RAIR-DTC are limited to FDA-approved tyrosine kinase inhibitors (TKIs) (Satapathy and Bal, 2022). However, the use of TKIs is associated with a variety of adverse events, such as cardiotoxicity, hematologic toxicity, fatigue, skin reactions, etc., which can limit patients’ daily activities, reduce compliance, and lead to high treatment withdrawal rates (Chrisoulidou et al., 2015). Timely identification and intervention for MTC are crucial, as patients diagnosed with early-stage MTC exhibit a five-year survival rate of 90 percent. Nevertheless, the overall survival in all stages of MTC remain disheartening, with a five-year survival rate of less than 40 percent (Bhoj et al., 2021). The objective response rates of cytotoxic chemotherapy are suboptimal, while targeted therapy and immunotherapy demonstrate limited effectiveness (Angelousi et al., 2022). Due to genetic factors and the lack of effective and safe medical therapy, guidelines from both the American Thyroid Association and the British Thyroid Association recommend total thyroidectomy for MTC (Yang et al., 2022). As the deadliest thyroid malignancy, ATC is highly metastasis and has long lacked reliable treatment, with a mortality rate approaching 100% (Haddad et al., 2022). ATC patients often do not respond well to conventional treatment, including radio-iodine ablation, chemotherapy, and external-beam radiotherapy (Saini et al., 2019).

Thyroid cancer survivors may necessitate ongoing surveillance, remedial therapy (surgery or RAI therapy), and thyroid hormone supplementation. Additionally, they may contend with the adverse consequences of thyroid hormone fluctuations, surgical complications, or side effects linked to RAI administration, as well as encounter enduring physical, psychosocial, and economic challenges (Lubitz and Sosa, 2016; Hedman et al., 2017). Standard treatments frequently entail complications, including postoperative issues such as damage to the parathyroid glands, recurrent laryngeal nerve, and laryngeal nerve, which significantly impact patients’ quality of life (Nagel et al., 2022). The administration of RAI entails immediate risks such as nausea, vomiting, insomnia, loss of taste, swelling and pain in the salivary glands. Additionally, there are potential long-term complications including recurring sialadenitis accompanied by dry mouth, oral pain, dental caries, pulmonary fibrosis, nasolacrimal outflow tract obstruction, and a secondary primary malignancy (Lee, 2010).

Furthermore, the long-term postoperative survival of TC patients poses a significant financial burden on both individuals and society. A comprehensive analysis of stacked cohorts in the United States (Lubitz et al., 2014), spanning from 1985 to 2013, revealed that the total societal cost of care for TC patients diagnosed after 1985 amounted to $1.6 billion in 2013. Notably, the expenses associated with survivor surveillance and non-surgical deaths resulting from thyroid cancer care accounted for 59% of the overall cost, surpassing the expenditures related to diagnosis, surgery, and adjuvant treatment for newly diagnosed patients, which constituted 41% of the total cost. A study of 52,012 adult thyroid cancer patients undergoing thyroid surgery (Sahli et al., 2021) suggests that there is a more cost-effective shift in thyroid surgery practice, such as an increase in outpatient surgery, but the cost continues to increase by 4.3% per year. Mongelli (Mongelli et al., 2020) et al. included 1,743 TC survivors for a questionnaire. The findings of the research indicated that 23.7% of the participants had depleted a significant portion or the entirety of their savings. 15.1% of the respondents resorted to borrowing funds from acquaintances or family members, and a smaller percentage of 3% declared bankruptcy. Moreover, 12% of the individuals reached the maximum limit on their credit cards, and 4.4% were compelled to seek additional loans or mortgages, and 15.9% reported being contacted by debt collection agencies. They suggested that TC survivors had relatively high bankruptcy filings, and that various forms of financial strain were linked to diminished health-related quality of life in this population. Based on the current increase in thyroid cancer incidence, it is projected that the total societal cost of care for TC patients will reach a sum of $3.5 billion by the year 2030 (Lubitz et al., 2014).

Patients often require lifelong monitoring, and cancer recurrence also carries a psychological burden. They may confront chronic psychiatric issues such as health anxiety (Zoltek et al., 2022). The apprehensions associated with TC are particularly pronounced among younger survivors, as well as those who have been confirmed or suspected relapses. Various factors, such as age, sex, educational attainment, marital status, ethnicity, transition to parenthood, time and disease severity after thyroid cancer diagnosis, have been found to be linked to the level of concern (Bresner et al., 2015; Papaleontiou et al., 2019). Consequently, the importance of thyroid cancer therapy optimization should not be overshadowed by the relatively high survival rate. The identification of cost-effective and convenient drug and effective therapeutic targets is of utmost urgency, which helps to reduce the physical, economic, psychological pain and social burden of patients.

## 3 Promising natural products for the treatment of thyroid cancer

### 3.1 Quercetin

Quercetin, a polyphenolic flavonoid that is commonly present in numerous foods and herbs, constitutes a regular component of a typical dietary intake. It has been employed for the purpose of mitigating or averting an array of ailments, encompassing cardiovascular disease, cancer, diabetes, neurological disorders, obesity, allergic asthma, and atopic diseases (Ulusoy and Sanlier, 2020). Quercetin can diminish PTC cell (BCPAP) viability through the initiation of apoptosis, reduction of cell adhesion and migration, induction of partial mesenchymal-to-epithelial transformation phenotype, and stimulation of NIS expression and RAI uptake (Goncalves et al., 2021; Sun et al., 2022). The significance of NIS expression, localization, and function in RAI treatment is widely acknowledged, thereby indicating the potential therapeutic value of quercetin in RAI treatment. NAG-1, a member of the TGF-β superfamily cytokine, exists in two primary forms: precursor and mature (Baek and Eling, 2019). These two forms of NAG-1 exhibit distinct activities in relation to cancer: the precursor type demonstrates anticancer properties, while the mature type promotes cancer development during tumorigenesis (Min et al., 2016). In comparison to normal controls, thyroid cancer tissue expresses higher levels of mature NAG-1, whereas normal thyroid tissue expresses higher levels of precursor NAG-1. When applied to PTC cell lines, quercetin induces the expression of precursor NAG-1 but not mature NAG-1, leading to apoptosis and cell cycle arrest (Hong et al., 2021). Bromodomain and extra-terminal (BET) proteins play a crucial role as epigenetic readers in the development of cancer. BET inhibitors have been developed as anticancer drugs, and their limited monotherapy activity and drug resistance have led to the attention of combination therapy (Stathis and Bertoni, 2018). hnRNPA1 and BET protein BRD4 are co-expressed in the human thyroid gland. Notably, the targeted intervention of hnRNPA1 with quercetin has been found to enhance the efficacy of BET inhibitors in thyroid cancer cells (K1 and 8505c) (Pham et al., 2019). Solafenib, a multi-kinase inhibitor possessing antiangiogenic properties, has been granted approval for the treatment of DTC (Cabanillas et al., 2016). However, the frequent dose-dependent side effects of sorafenib in clinical trials impede its efficacy in suppressing cancer. *In vitro* studies have demonstrated that co-administration of quercetin with sorafenib can mitigate the required anticancer dose against thyroid cancer cells (Celano et al., 2020).

### 3.2 Genistein

Genistein, a 7-hydroxyisoflavone, is commonly recognized as an angiogenesis inhibitor, phytoestrogen and insect repellent. Its primary source is soybeans and soy products. It exerts inhibitory effects on PTC cell proliferation, induces cell death and cell cycle arrest, and counteracts epithelial-mesenchymal transition trends by preventing nuclear translocation of β-catenin (Zhang et al., 2019). As previously mentioned, certain medullary thyroid carcinomas (MTCs) arise due to gain-of-function mutations in the RET proto-oncogene, which encodes the transmembrane tyrosine kinase receptor. *In vitro* evaluation of MTC cells by tyrosine kinase inhibitors, genistein effectively inhibits cell growth and RET tyrosine kinase activity in a dose-dependent manner (Cohen et al., 2002). A case-control study comprising 387 histologically confirmed cases of thyroid cancer and 433 normal control populations suggests that adequate intake of genistein is protective in women with thyroid cancer (>1 cm) (Wang et al., 2020).

### 3.3 Apigenin

Apigenin, a trihydroxyflavonoid, exhibits low toxicity and is frequently present in olive oil, sage, marjoram, and other sources. It possesses a diverse range of advantageous biological properties, encompassing antitumor, antioxidant, anti-inflammatory, antiviral, and other activities (Xu X. et al., 2020a). By stimulating the production of reactive oxygen species, apigenin induces DNA damage, resulting in G2/M cell cycle arrest and subsequent autophagic cell death. This mechanism effectively inhibits the activity of papillary thyroid cancer cells (Zhang L. et al., 2015a). And apigenin can induce apoptosis in ATC cells through c-Myc-mediated apoptosis, accompanied by phosphorylation of p53 and p38 (Kim et al., 2013). The inhibitory effect of TGF-β on radioactive iodine uptake, achieved through the downregulation of NIS, can be counteracted by the administration of apigenin. This suggests that apigenin holds potential as a dietary supplement to improve the therapeutic efficacy of radioactive iodine at the margins of aggressive tumors, thereby mitigating the incidence of metastatic events (Lakshmanan et al., 2015).

### 3.4 Luteolin

Luteolin is a tetrahydroxyflavonoid with antioxidant, anti-inflammatory, anticancer, and immunomodulatory activities. It was initially employed in the production of dyes derived from thyme, olive oil, rosemary, artichoke and oregano (Lopez-Lazaro, 2009). Luteolin can reduce the expression of BRAF-activated BANCR, further downregulate TSHR, and exert anti-PTC and FTC effects *in vitro* (Liu et al., 2017).

### 3.5 Chrysin

Chrysin is a dihydroxyflavonoid present in honey, propolis, passionflower, and Indian trumpet flower, and is widely acknowledged for its neuroprotective, anti-inflammatory, antioxidant, and anticancer properties (Zhang Z. et al., 2015b). The functional activity of Notch1 induced by chrysin is confirmed through a luciferase reporter gene assay incorporating the C promoter-binding factor 1 binding site. The novel Notch1 activator chrysin inhibits tumor growth in ATC both *in vitro* and *in vivo*. Compared with the control group, oral administration of chrysin leads to a significant average inhibition of ATC xenograft growth by 59%. Additionally, the median tumor progression time in the group treated with chrysin is approximately twice as long as that observed in the control group of mice with tumors (Yu et al., 2013).

### 3.6 Myricetin

Myricetin is a hexahydroxyflavonoid commonly found in wine, oranges, and other sources, which exhibits cytotoxic effects on PTC cells and ATC cells. Furthermore, it induces dose-dependent DNA agglomeration, upregulates the activation of the caspase pathway, and enhances the expression of bax/bcl2, thereby inducing apoptosis (Ha et al., 2017; Jo et al., 2017). In a comparative study examining the variances in growth and iodide content among various natural flavonoids (kaempferol, apigenin, luteolin, myricetin) within human Na^+^/I^−^ homologous transfected FTC cell lines, myricetin exhibited the unique characteristic of enhancing iodization intake while concurrently reducing iodine efflux. This finding implies a potentially superior therapeutic efficacy in TC RAI therapy (Schroder-van der Elst et al., 2004).

### 3.7 Resveratrol

Resveratrol is a stilbene compound predominantly found in red grapes, red wine, cranberries, strawberries, red currants, mulberries, and peanuts (Neveu et al., 2010). This widely recognized NP exhibits a diverse range of biological activities, including anti-inflammatory, antioxidant, anticancer, anti-aging, anti-diabetic, and anti-obesity activities (Bird et al., 2017; Wu SX. et al., 2022a). DMD encompasses three different genotoxic carcinogens, namely, diethylnitrosamine (DEN), dihydroxy-di-N-propylnitrosamine (DHPN), and N-methyl-N-nitrosourea (MNU). Animal models of TC induced by the carcinogen DMD are suitable for investigating potential therapeutic agents capable of impeding the progressive cellular and molecular alterations of TC. The administration of resveratrol via intragastric and intraperitoneal injection has been shown to effectively mitigate the occurrence and severity of TC-related lesions. Furthermore, prolonged resveratrol treatment has demonstrated the potential to enhance the overall health of rats induced with DMD. Additionally, oral administration of resveratrol can achieve a similar therapeutic effect to intraperitoneal injection (Zheng et al., 2018a). Multiple studies have demonstrated that resveratrol has good inhibitory effects in PTC, ATC and MTC. Resveratrol exerts its effects by modulating the Ras-MAPKK-MAPK signaling pathway, leading to increased expression of p53, serine phosphorylation of p53, and p53-dependent apoptosis in PTC and FTC cell lines, thereby exerting tumor suppressor effects (Shih et al., 2002). The sialyltransferase (ST) family and the Hippo signaling pathway (Sekido, 2018) play a key role in cancer regulation, with ST further classified into ST3GAL, ST6GAL, ST6GALNAC, and ST8SIA. The findings from next-generation sequencing revealed a significant upregulation of ST6 beta-galactoside alpha-2,6-sialyltransferase 2 (ST6GAL2) mRNA expression in FTC cells (FTC133 and FTC238) compared with normal thyroid cells. Furthermore, *in vitro* experiments have demonstrated that this upregulation has a regulatory effect on tumor proliferation, migration and invasion. In *in vitro* and *in vitro* experiments, resveratrol significantly inhibits the occurrence and development of FTC by modulating the ST6GAL2-Hippo pathway (Xu G. et al., 2020b). Oxidative damage caused by the accumulation of reactive oxygen species in cells is one of the therapeutic effects of anticancer drugs, which is closely related to the chemical sensitivity of cancer cells. Resveratrol is able to increase reactive oxygen species production and oxidation-associated cytopathies in resveratrol-sensitive ATC cell line THJ-16T by activating the reactive oxygen species-mitochondrial signaling pathway (Zheng et al., 2018b). Moreover, resveratrol triggers Notch2-mediated apoptosis of MTC cells and suppresses the expression of neuroendocrine markers ASCL1 and CgA (Truong et al., 2011).

Resveratrol has been found to increase iodine capture in rat thyroid cell FRTL-5, which has an additive effect with TSH and can increase iodide influx and RIS protein levels in rats even in the absence of TSH (Sebai et al., 2010). However, it has been suggested that resveratrol has little effect on the proliferation and intracellular distribution of human normal thyroid cell lines (Nthy-ori 3-1). Additionally, while resveratrol may promote the redifferentiation of ATC cells and upregulation of NIS expression, it remains challenging to enhance ATC iodine uptake (Xiong et al., 2020). In fact, the existing literature on resveratrol and TC iodine uptake is limited, necessitating further research to address the conflicting findings.

Tretinoin is commonly employed in conjunction with radiation therapy for the treatment of aggressive thyroid cancer. However, retinoid-based differentiation therapy remains a topic of controversy (Courbon et al., 2006). The ATC cell line THJ-11T is not sensitive to tretinoin therapy. CRABP2 is considered a core player in the exertion of retinoic acid in tumor suppression (Yang et al., 2016), while resveratrol can upregulate thyroglobulin and cadherin E expression by activating CRABP2/RAL-mediated tumor suppressor signaling, thereby effectively reversing the resistance of THJ-11T to tretinoin (Li et al., 2018). The combination therapy of dabrafenib, an FDA-approved BRAF inhibitor, and trametinib, a MEK inhibitor, against BRAF^V600E^-mutated ATC (Subbiah et al., 2020), can extend survival to 12 months in 12 percent of patients (Subbiah et al., 2018). In contrast, resveratrol has a more potent anti-ATC effect compared to BRAF-MAPK-targeted drugs (dabrafenib and trametinib) by simultaneously inhibiting the BRAF-MAPK and STAT3 signaling pathways in ATC cell lines with BRAF fusion or point mutation (Lu et al., 2022). The activation of JAK2/STAT3 is thought to be a significant factor contributing to cancer cell resistance towards drugs targeting the MAPK pathway (Crispo et al., 2019). Resveratrol can effectively inhibit the signaling of STAT3 activated by dabrafenib and trametinib, suggesting that combining resveratrol with BRAF-MAPK-targeted agents could potentially enhance the efficacy of treatment for ATC. Furthermore, resveratrol can also modulate PI3K/AKT/mTOR signaling, increasing the sensitivity of anti-PTC cell lines to rapamycin (Bian et al., 2020). This non-toxic polyphenolic compound has potential value in improving the clinical management of lethal ATCs, especially those that develop drug resistance.

### 3.8 Curcumin

Turmeric, a plant frequently employed in South and Southeast Asian tropical regions for the production of curry powder, possesses a rhizome that holds significant value for culinary and medicinal applications. Curcumin, the most potent constituent within turmeric, exhibits diverse biological functions encompassing, anti-inflammatory, antibacterial, and anticancer properties (Shishodia et al., 2005). Curcumin dose-dependently inhibits the proliferation and migration of PTC cell line K1 cells (Tan et al., 2015), while also induces its apoptosis. This ability to resist apoptosis is attributed to the rapid production of significant levels of reactive oxygen species (ROS) and increased intracellular Ca^2+^ concentrations (Song et al., 2012). Curcumin has been observed to exhibit a dose-dependent inhibition of cell viability in the PTC cell lines B-CPAP and KTC-1. Additionally, it has been found to inhibit the JAK/STAT3 signaling pathway and elevate ROS levels to induce apoptosis (Zhang et al., 2016; Khan et al., 2020). Furthermore, curcumin can promote cell differentiation and enhance the expression of thyroid-specific transcription factors TTF-1, TTF-2, and PAX8, along with iodine metabolism proteins such as thyroid-stimulating hormone receptor, thyroid peroxidase and sodium iodide transporter. Significantly, curcumin has the ability to enhance NIS glycosylation and facilitate its membrane transport, resulting in notable enhancements *in vitro* radioactive iodine uptake. And it can enhance radioiodine sensitivity by inhibiting the PI3K-AKT-mTOR signaling pathway (Zhang L. et al., 2021b). RAI therapy, widely employed in thyroid cancer, frequently induces impaired salivary gland dysfunction. The combination treatment of RAI and curcumin showed evidence of tissue remodeling compared with the control group, and resulted in a higher count of salivary epithelial cells, salivary duct cells, endothelial cells and myoepithelial cells, thereby ameliorating RAI-induced salivary gland dysfunction in mice (Kim et al., 2019). The overexpression of HO-1 has been shown to reduce cell viability and potentially activate ferroptosis signaling pathway, while curcumin has the ability to modulate the expression of HO-1 in the ferroptosis pathway, to thereby inhibit FTC growth (Chen et al., 2023).

The aggressive nature of ATC is primarily attributed to the presence of cancer stem cell (CSC) phenotype (Lee et al., 2022). The efficacy of cytostatic compounds is largely compromised due to the multidrug resistance mechanism driven by the CSC phenotype. In intervention experiments conducted on the ATC cell line CAL-62, curcumin could significantly inhibit the spheroid formation and cell motility in Matrigel, inhibit the accumulation of G0/1 phase cells and the oxidative stress index, and alter the invasion behavior of ATC cells through the inhibition of the CSC phenotype (Kocdor et al., 2019). Curcumin synergistically enhances the anticancer activity of cisplatin in PTC cells and cancer stem cell-like cells by targeting STAT3 (Khan et al., 2020), and can also enhance the antitumor activity of docetaxel in ATC cells by interfering with NF-κB and COX-2 (Hong et al., 2014). This suggests that the combination of curcumin and chemotherapy drugs may provide better therapeutic effect.

### 3.9 Nobiletin

Nobiletin is a hexamethoxyflavone with various activities such as enhancing circadian rhythm (He et al., 2016), antagonizing metabolic syndrome, inhibiting tumor (Chen et al., 2022), and treating liver ischemia (Dusabimana et al., 2019). It is commonly derived from citrus peel but can also be found in Chinese herbal medicines such as *Centipedae Herba, Citrus Reticulata, Tripterygii Radix*. Bioinformatics analysis and cell assays showed that nobiletin suppressed the proliferation and migration of a PTC cell line (B-CPAP) by modulating the PI3K/AKT signaling pathway (Du et al., 2023). The viability of ATC cell lines (T235 and T238) with the intervention of nobiletin, was observed to decrease in a dose-dependent manner, but the cell cycle was not affected. Moreover, at a concentration of 100μM, nobiletin was as effective in reducing ATC cell viability as conventional drugs such as cisplatin, while nobiletin was less toxic to normal thyroid cells (Sousa et al., 2020).

## 4 Outlook and summary

NPs and their derivatives have consistently demonstrated remarkable efficacy against a range of diseases (Hassan et al., 2022), particularly cancer (Memariani et al., 2021) and infectious diseases. Nevertheless, the advancement and clinical implementation of these NPs encounter numerous challenges, necessitating the overcoming of technical barriers in screening, isolation, characterization, and optimization. Unmodified NPs may exhibit various deficiencies in absorption, distribution, metabolism, excretion, and toxicity (Alexander et al., 2016). Fortunately, recent advances in the field of science and technology have presented a multitude of opportunities for the development of NP drugs, such as improved omics analysis tools (Wolfender et al., 2019), genome mining and engineering techniques (Kayrouz et al., 2020), microbial culture systems, and novel drug delivery systems (Lou et al., 2023). Drug nanotechnologies have proven to be one of the most efficient and reliable delivery systems due to their ability to enhance solubility, absorption, pharmacokinetics, bioavailability and provide toxicity protection (Alexander et al., 2016). Nanoparticles loaded with nobiletin have a small and uniform size and show beneficial potential in enhancing colloidal stability and averting premature drug seepage. These nanoparticles possess superior capabilities in inducing apoptosis in tumor cells and inhibiting metastasis in both human non-small cell lung cancer and human fibrosarcoma, surpassing the efficacy of naked drug formulations (Wu D. et al., 2022b). Injectable targeted nanoparticles developed for advanced hepatocellular carcinoma can effectively transport curcumin and resveratrol to hepatoma cells (Zheng et al., 2022). This novel formulation can reduce the dose of the drug and increase the bioavailability of the encapsulated drug. A significant increase in the concentration of the drug around the tumor is often accompanied by a favorable therapeutic effect and negligible adverse effects. Koppolu et al. (2012) developed a temperature-sensitive silane-coupled iron oxide nanoparticles as targeted drug delivery vehicles for treatments of ATC, and *in vitro* tests were performed with doxorubicin. Consequently, despite the numerous obstacles, the significance of nanoparticles in drug development and the optimization of novel drugs remains substantial.

This article provides an overview of plant-derived NPs that exhibit promising potential in the treatment of TC. Nevertheless, there are some weaknesses in this review. First, although these components have shown good results in cell and animal experiments in intervening PTC, only a small number of relevant clinical studies have been retrieved, and there is a lack of evidence from a large number of clinical studies to demonstrate the safety and therapeutic potential of the selected NPs in TC patients. However, there are studies that show the safety of the mentioned NPs in cells, animals, healthy people. High doses of quercetin in animal studies may lead to enhanced nephrotoxic effects, and limited data from human intervention studies have not been shown to have an adverse effect on kidney function (Andres et al., 2018). Nano-genistein is well tolerated by animals, and no toxic effects are found in animals with an intervention of nano-genistein doses up to 400 mg/kg/day for up to 7 days per week for 20 weeks (Kaytor et al., 2023). *In vitro* studies apigenin has no toxic effect on some normal cells, such as prostate epithelial cells and hepatocytes (Ahmed et al., 2021), and computer prediction tools have shown that apigenin is not hepatotoxic or skin sensitized (Hossain et al., 2023). Although luteolin has been reported to cause cytotoxicity in primary rat hepatocytes, this is time- and dose-dependent (Yao et al., 2023). *In vivo* experiments on zebrafish eggs showed that the toxicity of chrysin loaded poloxamer micelles was dose-effective, which are safe for the growth of zebrafish embryos at drug doses of 10 ng/mL or less (Sassa-Deepaeng et al., 2016). Myricetin has been acknowledged as generally safe, as evidenced by the absence of mortality in mice even at high doses of 1,000 mg/kg administered intraperitoneally, but the release of reactive oxygen species at pH above 7.4 may cause toxic effects on biomolecules (Rahmani et al., 2023). Resveratrol is considered safe. No serious adverse events were detected on clinical, biochemical, or hematologic measures during the intervention and 2-week follow-up phases in healthy populations, with the most common toxicity being gastrointestinal toxicity (Patel et al., 2011). The safety, tolerability, and non-toxicity of high-dose curcumin in healthy people have been demonstrated through clinical trials (Gupta et al., 2013). In a randomized, placebo-controlled, double-blind, crossover study (Ito et al., 2023), no associated serious adverse events were observed in nocturia patients taking a mixture of nobiletin and tangeretin. There was a lack of statistically significant impact observed on blood pressure or heart rate, and no clinically aberrant results were identified in hematological or biochemical parameters. In general, the safety of NPs in TC needs to be continuously explored.

Second, this review selects only some NPs derived from plants, especially herbal medicines. Among the NPs involved in this paper, it is obvious that resveratrol and curcumin have more preclinical studies on TC and have better research accumulation compared with other NPs. The Clinicaltrials.gov currently has 329 clinical trials for curcumin, of which 86 are anti-cancer, and resveratrol has 209 clinical trials, of which 19 are anti-cancer. Most of these NPs have inhibitory effects on TC *in vitro* and *in vivo*. Quercetin, apigenin, myricetin, and curcumin may have potential therapeutic value in promoting iodine uptake in TC, while resveratrol is controversial for the redifferentiation of TC. Quercetin, resveratrol, and curcumin have performed well in combination therapy and may help alleviate the side effects and resistance of some approved drugs. In addition, what is more interesting is the protective effect of genistein in human studies. There are relatively few studies on luteolin, chrysin, myricetin, and nobiletin in TC, and they have good therapeutic potential, which deserve more attention. And the review lacks information on NPs derived from animals, microorganisms, and marine organisms.

Third, there are still many gaps to be filled in the development of novel drug delivery systems for TC. Existing studies have focused on ATC and lack attention to other pathological types. As mentioned above, resveratrol has more basic research, and we can pay attention to related nano-formulations, such as liposomes, polymer nanoparticles, lipid nanocarriers and inorganic nanoparticles, etc. (Sarfraz et al., 2023), which will open up new ways to explore the nano-system development of other NPs. Application of these recommendations may accelerate the clinical translation of NPs in TC treatment, providing TC survivors with a wider range of clinical treatment alternatives.
